# The Use of Tranexamic Acid to Prevent Postpartum Hemorrhage

**DOI:** 10.1111/jmwh.13101

**Published:** 2020-05-19

**Authors:** Ruth T. Mielke, Sarah Obermeyer

**Affiliations:** ^1^ California State University, Fullerton Fullerton California; ^2^ Eisner Pediatric and Family Medical Center Los Angeles California; ^3^ Azusa Pacific University Azusa California

**Keywords:** antifibrinolytic, obstetric hemorrhage, postpartum hemorrhage, tranexamic acid

## Abstract

Tranexamic acid (TXA) is an antifibrinolytic pharmacologic agent with demonstrated effectiveness for reducing the incidence of death from blood loss following trauma and major surgery. In intrapartum care, TXA is being used in in conjunction with uterotonic agents to treat postpartum hemorrhage (PPH). Based on the findings of the WOMAN trial that found TXA reduced maternal death due to PPH, the World Health Organization recommends that TXA be part of the standard comprehensive PPH treatment package, and US professional organizations recognize its use as adjunctive treatment for PPH. Evidence suggests that TXA used prophylactically in the setting of cesarean birth may decrease blood loss and the incidence of PPH. There is limited evidence for prophylactic use of TXA in women of all risk categories following vaginal birth but prophylactic use in women who have an a priori risk for PPH is being investigated. This article presents a case in which a midwife identifies a woman in active labor who has significant risk factors for PPH. In consultation with the collaborating obstetrician, TXA is given early during the third stage of labor in addition to the recommended components of active management for the purpose of preventing PPH.

## CASE SUMMARY

*E.B. is a 20‐year‐old gravida 3 para 2 at 39 3/7 weeks’ gestation admitted to the labor and birth unit in early active labor. Her prenatal course was complicated by iron deficiency anemia with an antenatal hemoglobin of 8.5 g/dL and hematocrit of 27.5%, which was identified one month prior to admission for labor and birth. She endorsed an inability to tolerate oral iron supplements. Her perinatal history was significant for 2 term vaginal births, with the second birth complicated by a postpartum hemorrhage (PPH) that occurred on arrival to the postpartum unit 2 hours after birth. She required a blood transfusion*.

*On admission, her hemoglobin was 8.2 g/dL, hematocrit 27.0%; mean corpuscular hemoglobin 26.0 pg (normal range, 27.0‐33.0); mean corpuscular volume 79.5 fL (normal range, 80.00‐100.00), and platelets 278,000 μL. Based on her risk factors, it was determined that she was at high risk for PPH. The midwife consulted with the collaborating obstetrician and they decided to have additional uterotonics available in addition to those used as standard elements of active management of third stage of labor (AMTSL). In addition, they ordered a type and crossmatch for 2 units of packed red blood cells. The use and timing of tranexamic acid (TXA) administration was also discussed. Based on her history, specifically of later onset PPH, TXA 1 g diluted in 100 mL of lactated ringers (LR) solution was given intravenously at the time of the birth of the newborn's anterior shoulder. The newborn had Apgar scores of 8 and 9. Immediate mild uterine atony in third stage was noted, and per institution policy, E.B. was given oxytocin (30 units in 500 mL of LR solution [200 mL bolus followed by 41 mL/h]), then methergine (0.2 mg given intramuscularly). The total quantitative blood loss was 450 mL. Twenty‐four hours postpartum, E.B.’s hemoglobin was 8.0 g/dL and hematocrit was 26.2%. She did not have orthostatic symptoms and was counseled on increasing dietary iron intake. She was discharged on the second postpartum day with instructions pertinent to anemia*.

## INTRODUCTION

Hemorrhage is the leading cause of maternal morbidity and mortality worldwide. In the United States, 11% of pregnancy‐related deaths were due to hemorrhage in the years 2011 to 2016.^1^ In 2 analyses of US hospital births, the incidence of PPH increased 26%, from 2.3% in 1994 to 2006 to 2.9% in 2012 to 2013.^2^, ^3^ In the context of community birth, 4.8% women in birth centers lost 1000 mL or more following birth, and PPH accounted for most postpartum transfers in planned home and birth center births.^4^, ^5^ Although the incidence of PPH varies depending upon the definition and criteria used, it is a complication that clinicians must be prepared to manage and, optimally, to prevent.

*Early PPH* has most recently been defined as “cumulative blood loss of 1000 ml or more of blood loss accompanied by sign and symptoms of hypovolemia within 24 hours following the birth process.”^6^ Worldwide, approaches to PPH are dictated by resource availability and include mechanical means (eg, bimanual compression, tamponade, antishock garments), replacement of fluid and blood products, embolization, and pharmacologic agents. In the United States, uterotonic agents are most often used for prevention and treatment of PPH. Oxytocin is the first‐line agent, and supplemental uterotonics used include methylergonovine (Methergine) and prostaglandins such as carboprost tromethamine (Hemabate) and misoprostol (Cytotec).^7^, ^8^

Historically, most drugs used to treat PPH were uterotonics with a mechanism of action that promoted uterine contraction, as uterine atony was presumed to be the underlying cause. However, the pathophysiology of severe PPH includes coagulopathy, and more recently, TXA, with its antifibrinolytic properties, is increasingly being used worldwide to treat PPH. Thus, a brief review of the relevant physiology is in order.

### Hypercoagulability During Pregnancy

Pregnancy is characterized by physiologic alterations in coagulation and fibrinolytic pathways. During pregnancy, there is (1) an increase in clotting factors (I, II, VII, VIII, IX, and XII), (2) a decrease in anticoagulation activity (protein S and activated protein C) and (3) a decrease in fibrinolysis resulting in a hypercoagulable state.^9^ This hypercoagulable state increases throughout pregnancy, peaking at term, and continues in the immediate postpartum period.^10^, ^11^ During the stress of labor, clotting factors further increase, and fibrinolytic activity decreases. After the separation of the placenta from the decidua, myometrial contractions occur, platelet aggregation at the placental site increases, and there is a release of coagulation factors that results in reduction of bleeding.^10^ The balance between coagulation and fibrinolysis maintains an intact vascular system, which diminishes the risk of PPH.

Hemostasis is the typical response to blood vessel or tissue injury.^12^, ^13^ Normally, damage to tissue results in activation of tissue factor. Tissue factor activates the clotting cascade, resulting in high levels of thrombin and stimulation of the intrinsic clotting pathway. Activation of thrombin in the clotting cascade leads to circulating fibrinogen being enzymatically converted into fibrin, which is the basis of clot formation. To counterbalance clotting, formation of fibrin stimulates the fibrinolytic system. The fibrinolytic cascade converts plasminogen, which is present in circulation and fibrin clots into plasmin. Plasmin is the primary enzyme that breaks down clots.

However, when blood loss is severe, the coagulation system and fibrinolysis can be severely dysregulated.^14^ In the event of severe PPH, shock‐related tissue hypoxia can lead to the release of excessive tissue factor from damaged cells, which is the primary driver that causes an imbalance between the coagulation and fibrolytic systems. In addition, during excessive bleeding, circulating fibrinogen and platelets are rapidly depleted via both coagulation and blood loss, which prevents further formation of fibrin clots.^15^, ^16^ The imbalance of the coagulation and fibrinolytic systems results in disseminated intravascular coagulation.^17^ Thus, the antifibrinolytic properties of TXA make this agent an ideal option for treatment of PPH.

## TRANEXAMIC ACID

TXA is a synthetic derivative of the amino acid lysine that acts by blocking plasminogen‐binding sites and inhibiting the proteolytic function of plasmin, thereby preventing fibrin degradation.^18^, ^19^ In 1962, Okamoto and Okamoto reported their discovery that TXA reduced bleeding by inhibiting fibrinolysis.^18^ When clot breakdown (fibrinolysis) is inhibited, overall bleeding is reduced.

In 2010, the Clinical Randomisation of an Antifibrinolytic in Significant Haemorrhage 2 (CRASH‐2) randomized controlled trial (RCT) reported the effects of early administration of TXA on outcomes of death, vascular occlusive events, and the receipt of blood transfusion in adult trauma patients.^20^ In the 10,096 patients allocated to the TXA group compared with the 10,115 in the placebo group, all‐cause mortality was significantly reduced within the TXA group compared with the placebo group (14.5% vs 16.0% respectively; relative risk [RR], 0.91; 95% CI, 0.85‐0.97; *P* = .0035). The risk of death from bleeding was significantly reduced (4.9% vs 5.7%; RR, 0.85; 95% CI, 0.76‐0.96; *P* = .0077).^20^ The results showed that early administration of TXA to trauma patients reduced the risk of death from hemorrhage, with no apparent increase in fatal or nonfatal vascular occlusive events.^20^ As TXA has been demonstrated to reduce the need for blood product transfusions and mortality, off‐label clinical applications are used in trauma and surgery, gastrointestinal bleeding, and more recently, PPH.^21^, ^22^, ^23^ TXA, administered orally or intravenously, is currently approved by the Food and Drug Administration for treatment of women experiencing abnormal uterine bleeding.^24^

In the United States at present, the use of TXA does not take precedence over the primary steps recommended for managing PPH and therefore it is considered adjunctive therapy.^7^, ^8^ In contrast, the World Health Organization recommends initiation of TXA in all cases of PPH regardless of etiology.^25^ A summary of current professional organization guidelines is presented in Table 1.

**Table 1:** Professional Organization Guidelines for the Use of Tranexamic Acid for Postpartum Hemorrhage

**Organization**	**Indication**	**Timing**	**Dosing**
World Health Organization	TXA should be used in all cases of PPH, regardless of whether the bleeding is due to genital tract trauma or other causes. TXA should be part of the standard comprehensive PPH treatment package, including medical (uterotonics), nonsurgical, and surgical interventions in accordance with WHO guidelines or adapted local PPH treatment guidelines.	Use TXA within 3 h and as early as possible after onset of PPH. Do not initiate TXA more than 3 h after birth, unless being used for bleeding that restarts within 24 h of completing the first dose.	Fixed dose of 1 g in 10 mL (100 mg/mL) IV at 1 mL/min (ie, administered over 10 min). Second dose of 1 g IV if bleeding continues after 30 min or if bleeding restarts within 24 h of completing the first dose.
American College of Obstetricians and Gynecologists	TXA should be considered in the setting of PPH when initial medical therapy fails.	Earlier use is likely to be superior to delayed treatment; benefit primarily in women treated sooner than 3 h from the time of birth.	Dosage not specified but refers to that which was used in WOMAN trial (1 g administered IV).
California Maternal Quality Care Collaborative	TXA is adjunctive treatment and *not* a primary treatment for PPH. The placement of TXA in the facility's hemorrhage guideline will depend on local resources.	Timing not specified but refers to WOMAN trial that “clearly demonstrated” that TXA is most effective when given within 3 h of hemorrhage diagnosis, hence the recommendation that it be considered relatively early in the hemorrhage guideline.	1 g IV over 10 min with a second 1‐g dose administered at 30 min if the bleeding persists; may be repeated once.

Abbreviations: IV, intravenously; PPH, postpartum hemorrhage, TXA, tranexamic acid; WHO, World Health Organization.

Source: Adapted from World Health Organization,^25^ American College of Obstetricians and Gynecologists,^7^ and California Maternal Quality Care Collaborative.^8^

Correct dose, timing, and method of TXA administration are critical. When TXA is used to treat PPH, it should be given as soon as possible (within a 3‐hour window) after the onset of bleeding and in conjunction with uterotonic medications if uterine atony is suspected.^7^, ^8^, ^25^ The recommended dose for treatment of PPH is 1 g administered over 10 minutes (Tables 1 and 2).^8^, ^25^ The onset of action for TXA when administered intravenously is 5 to 15 minutes. Very little of the total TXA dose is metabolized, resulting in more than 95% of the dose being excreted unchanged via the renal system. The half‐life is approximately 2 hours, and nearly all (90%) of the drug is excreted within 24 hours of administration.^19^

**Table 2:** Tranexamic Acid: Clinical Guidance for Treatment of Postpartum Hemorrhage

TXA should always be readily available in the birth and postpartum areas of facilities providing emergency perinatal care.
TXA should be initiated within 3 h after birth.
TXA should be administered at a fixed dose of 1 g in 10 mL (100 mg/mL) IV at 1 mL/min (ie, administered over 10 min), with a second dose of 1 g IV if bleeding continues after 30 min or if bleeding restarts within 24 h of completing the first dose.
TXA should be administered slowly as an IV injection over 10 min because bolus injection carries a potential risk of transient lowering of blood pressure.
TXA for injection may be mixed with most solutions for infusion, such as electrolyte solutions, carbohydrate solutions, amino acid solutions, and dextran solutions, and can be administered through the same IV cannula used for IV hydration or uterotonic administration. TXA should not be mixed with blood for transfusion, solutions containing penicillin, or mannitol.
TXA should not be administered to women with a clear contraindication to antifibrinolytic therapy, (eg, a known thromboembolic event during pregnancy, history of coagulopathy, active intravascular clotting, or known hypersensitivity to TXA).

Abbreviations: IV, intravenous(ly); TXA, tranexamic acid.

Source: Adapted from World Health Organization,^25^ American College of Obstetricians and Gynecologists,^7^ and California Maternal Quality Care Collaborative.^8^

The dose of TXA may be repeated once after 30 minutes if bleeding continues.^8^ Because TXA is excreted primarily through the kidneys, individuals with renal impairment may need a dosage adjustment. During pregnancy, the kidneys function at a higher demand, with glomerular filtration rate increasing by 45% to 50% and renal plasma flow increasing because of the effects of progesterone.^26^ Therefore, if an individual is diagnosed with a hypertensive disorder of pregnancy, diabetes, or other conditions that reduce kidney function, this should be considered when choosing a dose.

Given the action of TXA in preventing clot degradation, a key concern has been the potential risk for thromboembolism. Adverse effects include venous thromboembolic events, such as deep vein thrombosis and pulmonary embolism, stroke, myocardial infarction, renal failure or dysfunction, seizures, and mild gastrointestinal symptoms (nausea, vomiting, diarrhea).^27^ However an increased incidence of thromboembolism has not been found in studies of TXA for patients with trauma or PPH.^20^, ^21^, ^22^, ^23^ In 2019, a systematic review and meta‐analysis (268 studies; n = 38,978) by Yates et al reported that TXA administered perioperatively did not increase risk of adverse events compared with placebo or no intervention (RR, 1.05; 95% CI, 0.99‐1.12).^27^ Of note, 16 of the studies included addressed TXA use during cesarean birth.

## TRANEXAMIC ACID FOR THE TREATMENT OF POSTPARTUM HEMORRHAGE

Uterine atony is the cause of 70% to 80% of PPH cases. Less common causes include retained placental tissue, genital lacerations, and inherent or acquired clotting conditions.^8^ Therefore, the initial approach to the treatment of PPH is to address the underlying cause (eg, uterotonics for atony, extraction of retained tissue, or surgical repair of laceration). In 2017, the World Maternal Antifibrinolytic (WOMAN) trial reported the effects of TXA in women with a clinical diagnosis of PPH.^23^ In 20,060 women experiencing PPH (defined as clinically estimated blood loss of ≥500 mL after vaginal birth, ≥1000 mL after cesarean, or any blood loss suggestive of hemodynamic instability), TXA significantly reduced the incidence of death due to hemorrhage in the treatment group when compared with the control group (1.5% vs 1.9%; RR, 0.81; 95% CI, 0.65‐1.00; *P* = .045). All other causes of death and rate of hysterectomy did not differ significantly between groups.^23^ In addition, there were 33% fewer laparotomies done to control bleeding in women having either cesarean or vaginal births following TXA use.^23^

The findings of the WOMAN trial prompted the World Health Organization to update their treatment recommendations for PPH to include TXA as part of the standard care for women with PPH following vaginal or cesarean birth regardless of etiology.^25^ In limited resource settings, the low cost and ease of storage and administration of TXA are important factors in the context of PPH treatment.^25^, ^28^ In high‐resource settings where trauma care and surgery already occur, TXA is also readily accessible in labor and birth units.^8^ A recent study done in the United States suggested that TXA is highly likely to be cost saving when used routinely for PPH.^28^ However, at present, the use of TXA to treat PPH is considered to be adjunct treatment and not an initial treatment of PPH in high‐resource settings.^7^, ^8^

## TRANEXAMIC ACID FOR THE PREVENTION OF POSTPARTUM HEMORRHAGE

Best practice for prevention of PPH is AMTSL, with the use of uterotonics, specifically oxytocin, as the most critical element.^7^, ^29^ Studies suggesting the use of TXA for prevention of PPH for women experiencing cesarean have been reported. A recent systematic review and metanalysis of 18 RCTs reported on the use of TXA given prior to cesarean to reduce blood loss.^30^ In most of the studies (n = 14), the guideline was to give 1 g of TXA 10 to 20 minutes before the cesarean. The review compared outcomes of women who received intravenous TXA for prevention of bleeding following cesarean, which included a total of 1764 women in the cesarean and 1793 women in the placebo group. Compared with the women in the placebo group, the women in the TXA group had a 60% reduction in the risk of mild to moderate PPH, defined as more than 400 mL (RR 0.40; 95% CI, 0.24‐0.65; 5 trials; n = 786). In addition, the women in the treatment group had a 68% reduction in the risk for severe PPH defined as greater than 1000 mL blood loss (RR, 0.32; 95% CI, 0.12‐0.84; 5 trials; n = 1850) and a 70% decrease in the need for red blood cell transfusion (RR, 0.30; 95% CI, 0.18‐0.49; 10 trials; n = 1873). There were no safety concerns about the use of the antifibrinolytic agent from the 18 RCTs analyzed.^30^ An older Cochrane review of 12 studies of TXA that assessed prevention of PPH for women undergoing elective cesarean reported similar reductions in the risk for PPH but noted an increase in minor side effects such as diarrhea, nausea, and vomiting with the use of TXA.^31^

Currently, a large RCT with anticipated enrollment of 11,000 women is underway to assess whether prophylactic TXA lowers the risk of PPH in women undergoing a cesarean birth.^32^ The large sample size and high quality of the study will help to address concerns of studies done to date, including (1) the difficulty in assessing the adverse effects of TXA, specifically thromboembolic events because of their rare occurrence, and (2) the lack of larger trials to adequately assess the effect of prophylactic TXA for women having a cesarean birth.

Research on the use of TXA to prevent PPH in women anticipating vaginal birth has been conducted, but results to date are less robust than the positive effects noted for women who have a cesarean birth.^33^ In a Cochrane review of the use of prophylactic TXA for women with vaginal and cesarean birth, 3 trials from Turkey, Iran, and China studied the effectiveness and safety of TXA in 832 women who had a vaginal birth.^31^, ^34^, ^35^, ^36^ One study was a prospective, double‐blinded, RCT in which women received a 1‐g infusion of TXA (n = 228) or 5% glucose (n = 226) at birth of the newborn anterior shoulder.^35^ Baseline characteristics, inclusive of risk factors for PPH, did not differ between the 2 groups. AMTSL was employed in both groups, including injection of 10 IU of oxytocin within 2 minutes of birth and controlled cord traction following birth. Estimated postpartum blood loss was significantly lower in the experimental group than in the placebo group (261.5 ± 146.8 mL vs 349.98 ± 188.85 mL, *P* < .001). Additionally, the incidence of PPH (>500 mL) was also lower in the experimental group compared with that in the placebo control group (1.8% vs 6.8% respectively; RR, 3.76; 95% CI, 1.27‐11.15; *P* = .01). No episode of thrombosis occurred in the women who received TXA. In another double‐blinded RCT, 120 women received either 1 g of intravenous TXA or placebo in addition to 10 IU oxytocin immediately after birth of the newborn.^36^ This study excluded women with risk factors for PPH (Table 3). The mean total blood loss was lower in the women given TXA compared with those given the placebo (519 ± 320 vs 659 ± 402 mL, *P* = .036) as was the measured blood loss from birth of the placenta to 2 hours postpartum (69 ± 39 vs 108 ± 53 mL, *P* <0.001). In addition, the frequency of blood loss greater than 1000 mL was lower in the TXA group (7% vs 18%, *P* = 0.048).^36^ These studies suggest that prophylactic TXA may decrease blood loss and PPH in populations at risk for PPH as well as in those without risk factors, but the studies conducted so far are limited by small sample size.

**Table 3:** Example of Postpartum Hemorrhage Risk Assessment Tool

**Low Risk**	**Medium Risk**	**High Risk**
Singleton pregnancy Fewer than 4 previous births Unscarred uterus Absence of history of postpartum hemorrhage	Prior cesarean or uterine surgery More than 4 previous births Prolonged use of oxytocin Intra‐amniotic infection Magnesium sulfate use Large uterine fibroids Multiple gestation	History of postpartum hemorrhage Hematocrit <30% Known coagulation defect Bleeding on admission Previa, accreta, increta, percreta Abnormal vital signs (tachycardia and hypotension)

Source: Adapted with permission from Gabel et al.^42^

Most recently, 3891 women in France who had a vaginal birth and received prophylactic oxytocin were randomized to receive prophylactic TXA or a placebo.^37^ The primary outcome, PPH (blood loss ≥ 500 mL), was not different in the 2 groups: 8.1% of 1921 women (TXA) and 9.8% of 1918 women (placebo; RR, 0.83; 95% CI, 0.68‐1.01; *P* = .07). Secondary outcomes such as severe PPH (blood loss ≥1000 mL), blood transfusion, and postpartum blood count indices did not differ significantly between the groups. With respect to safety of TXA, the incidence of thromboembolic events in the 3 months after birth did not differ between the TXA group and the placebo group (0.1% and 0.2%, respectively; RR, 0.25; 95% CI, 0.03‐2.24).^37^ Although the study identified women at risk for PPH secondary to a previous history of PPH, this subgroup was less than 5% of the sample. The study was not powered to perform analyses of use of TXA in women at risk for PPH, thereby limiting its findings to the use of TXA in prevention of PPH in vaginal birth in general and not for those at risk for PPH.

The use of TXA as a preventive measure in women at high risk for PPH is germane to this case. Maternal anemia is a risk factor for PPH (Table 3), and women with anemia have worse outcomes after PPH.^38^, ^39^ Furthermore, increasing severity of anemia is positively correlated with more severe adverse maternal outcomes.^39^ It is not completely clear why the risk of PPH is higher in women with anemia, but it is postulated that uterine atony may be likely due to impaired oxygen transport to the uterine muscle.^40^ A cohort study of women in Assam, India, reported that women with severe anemia (hemoglobin [Hgb] level <7 g/dL) had more than a 9‐fold higher odds of PPH (adjusted odds ratio [aOR], 9.45; 95% CI, 2.62‐34.05); women with moderate‐severe anemia (Hgb 7–9.9 g/dL) had 50% increase in the odds of PPH (aOR, 1.50; 95% CI, 0.80‐2.80), and if labor induction was performed, women with moderate‐severe anemia had 17‐fold increase in the incidence of PPH (aOR, 17.39; 95% CI, 3.73‐80.97).^39^ A study conducted in Norway also reported that the likelihood of severe PPH (>1500 mL) was more than 2 times higher (aOR, 2.20; 95% CI, 1.63‐3.15) in women with anemia (Hgb <9 g/dL).^41^ Currently, the WOMAN‐2 trial is an international, double‐blinded, RCT (estimated study completion date April 2022) designed to quantify the effects of TXA used prophylactically in women giving birth with moderate or severe anemia.^40^ Ten thousand women with moderate or severe anemia who have given birth vaginally will receive either 1 g of TXA or matching placebo by intravenous injection immediately (within 15 min) after the umbilical cord is clamped and/or cut.^40^ Therefore, beyond the current components of AMTSL, prophylactic use of TXA is being studied in women at particular risk of PPH after vaginal birth.

## CONCLUSION

PPH is a leading cause of maternal mortality and morbidity worldwide.^25^ Clinicians providing intrapartum care must be prepared not only to treat but, if possible, to anticipate and prevent PPH. Evidence supports use of AMTSL with uterotonics and cord traction as a means of decreasing postpartum blood loss in women of all risk categories in low‐ and high‐resource settings. There is high‐level evidence that TXA is useful for treatment of PPH when given less than 3 hours after birth and is best used with oxytocin but before using blood products. At the present, there is research evidence that supports the prophylactic use of TXA to reduce blood loss and PPH in women having a cesarean birth, but the evidence for use during vaginal birth has not been fully determined.

In the case of E.B., the midwife and obstetrician were concerned that E.B. was at particular high risk for PPH secondary to her history of PPH and the presence of anemia (hematocrit <30%). Furthermore, at her prior birth, she had an atypical presentation of PPH 2 hours after birth that required a blood transfusion. E.B. did not possess risk factors for venous thrombosis. By giving TXA with birth of the newborn's anterior shoulder, placental transfer of TXA was mitigated while also allowing the antifibrinolytic properties to take effect early in the third stage. This management illustrates how TXA can be used for prevention of PPH in individual circumstances.

## CONFLICT OF INTEREST

The authors have no conflicts of interest to disclose.

Continuing education units (CEUs) are available for this article. To obtain CEUs online, please visit www.jmwhce.org. A CEU form that can be mailed or faxed is available in the print edition of this issue.

## References

[1] Creanga AA , Syverson C , Seed K , Callaghan WM . Pregnancy‐related mortality in the United States, 2011‐2013. Obstet Gynecol. 2017;130(2):366‐373. 28697109 10.1097/AOG.0000000000002114 PMC5744583

[2] Callaghan WM , Kuklina EV , Berg CJ . Trends in postpartum hemorrhage: United States, 1994‐2006. Am J Obstet Gynecol. 2010;202(4):353.e1‐353.e6. 20350642 10.1016/j.ajog.2010.01.011

[3] Reale SC , Easter SR , Xu X , Bateman BT , Farber MK . Trends in postpartum hemorrhage in the United States from 2010 to 2014 [published online September 18, 2019]. Anesth Analg. 10.1213/ANE.0000000000004424. 10.1213/ANE.0000000000004424 31567319

[4] Cheyney M , Bovbjerg M , Everson C , Gordon W , Hannibal D , Vedam S . Outcomes of care for 16,924 planned home births in the United States: the Midwives Alliance of North America statistics project, 2004 to 2009. J Midwifery Womens Health. 2014;59(1):17‐27. 24479690 10.1111/jmwh.12172

[5] Stapleton SR , Osborne C , Illuzzi J . Outcomes of care in birth centers: demonstration of a durable model. J Midwifery Womens Health. 2013;58(1):3‐14. 23363029 10.1111/jmwh.12003

[6] Menard KM , Main KE , Currigan MS . Executive summary of the reVITALize initiative: standardizing obstetric data definitions. Obstet Gynecol. 2014;124(1):150‐153. 24901267 10.1097/AOG.0000000000000322

[7] Committee on Practice Bulletins‐Obstetrics . Practice bulletin no. 183: postpartum hemorrhage. Obstet Gynecol. 2017;130(4):e168‐e86. 28937571 10.1097/AOG.0000000000002351

[8] California Maternal Quality Care Collaborative . Tranexamic Acid (TXA) for Obstetric Hemorrhage. Stanford, CA: California Maternal Quality Care Collaborative; July 2017 https://www.cmqcc.org/resource/tranexamic-acid-txa-obstetric-hemorrhage. Accessed August 24, 2019

[9] King TL. Anatomy and physiology of pregnancy In: King TL, Brucker MC, Osborne K, Jevitt CM, eds. Varney's Midwifery. 6th ed. Burlington, MA: Jones & Bartlett Learning; 2019; 633‐662.

[10] Anderson CA , Avery MD. Anatomy and physiology during labor and birth In: King TL, Brucker MC, Osborne K, Jevitt CM, eds. Varney's Midwifery. 6th ed. Burlington, MA: Jones & Bartlett Learning, LLC; 2019:861‐880.

[11] Physiology of parturition. In: Coad J , Dunstall M. Anatomy and Physiology for Midwives. 3rd ed: London: Churchill Livingstone; 2012:317‐362.

[12] Chang JC. Hemostasis based on a novel ‘two‐path unifying theory’ and classification of hemostatic disorders. Blood Coagul Fibrinolysis. 2018;29(7):573‐584. 30063477 10.1097/MBC.0000000000000765

[13] Chapin JC , Hajjar KA. Fibrinolysis and the control of blood coagulation. Blood Rev. 2015;29(1):17‐24. 25294122 10.1016/j.blre.2014.09.003 PMC4314363

[14] Hayakawa M. Pathophysiology of trauma‐induced coagulopathy: disseminated intravascular coagulation with the fibrinolytic phenotype. J Intensive Care. 2017;5:14. 28289544 10.1186/s40560-016-0200-1 PMC5282695

[15] Lockwood CJ , Murk W , Kayisli UA , et al. Progestin and thrombin regulate tissue factor expression in human term decidual cells. J Clin Endocrinol Metab. 2009;94(6):2164‐2170. 19276228 10.1210/jc.2009-0065 PMC2690421

[16] Charbit B , Mandelbrot L , Samain E , et al. The decrease of fibrinogen is an early predictor of the severity of postpartum hemorrhage. J Thromb Haemost. 2007;5(2):266‐273. 17087729 10.1111/j.1538-7836.2007.02297.x

[17] Lockwood CJ , Paidas M , Murk WK , et al. Involvement of human decidual cell‐expressed tissue factor in uterine hemostasis and abruption. Thromb Res. 2009;124(5):516‐520. 19720393 10.1016/j.thromres.2009.07.017 PMC2787516

[18] Okamoto S , Okamoto U . Amino‐methyl‐cyclohexane‐carboxylic acid: AMCHA: a new potent inhibitor of the fibrinolysis. Keio J Med. 1962;11(3):105‐115.

[19] Pilbrant Å , Schannong M , Vessman J . Pharmacokinetics and bioavailability of tranexamic acid. Eur J Clin Pharmacol. 1981;20(1):65‐72. 7308275 10.1007/BF00554669

[20] CRASH 2 Trial Collaborators ; Shakur H , Roberts I , Bautista R , et al. Effects of tranexamic acid on death, vascular occlusive events, and blood transfusion in trauma patients with significant haemorrhage (CRASH‐2): a randomised, placebo‐controlled trial. Lancet. 2010;376(9734):23‐32. 20554319 10.1016/S0140-6736(10)60835-5

[21] Myles PS , Smith JA , Forbes A , et al. Tranexamic acid in patients undergoing coronary‐artery surgery. N Engl J Med. 2017;376(2):136‐148. 27774838 10.1056/NEJMoa1606424

[22] Roberts I , Coats T , Edwards P , et al. HALT‐IT– tranexamic acid for the treatment of gastrointestinal bleeding: study protocol for a randomised controlled trial. Trials. 2014;15:450. 25409738 10.1186/1745-6215-15-450 PMC4253634

[23] WOMAN Trial Collaborators . Effect of early tranexamic acid administration on mortality, hysterectomy, and other morbidities in women with post‐partum haemorrhage (WOMAN): an international, randomised, double‐blind, placebo‐controlled trial. Lancet. 2017;389(10084):2105‐2116. 28456509 10.1016/S0140-6736(17)30638-4 PMC5446563

[24] Bradley LD , Gueye NA. The medical management of abnormal uterine bleeding in reproductive‐aged women. Am J Obstet Gynecol. 2016;214(1):31‐44. 26254516 10.1016/j.ajog.2015.07.044

[25] World Health Organization . Updated WHO Recommendation on Tranexamic Acid for the Treatment of Postpartum Haemorrhage. Geneva, Switzerland: World Health Organization; 2017 https://apps.who.int/iris/bitstream/handle/10665/259379/WHO-RHR-17.21-eng.pdf;sequence=1. Accessed January 26, 2020. 29630190

[26] Cheung KL , Lafayette RA. Renal physiology of pregnancy. Adv Chronic Kidney Dis. 2013;20(3):209‐214. 23928384 10.1053/j.ackd.2013.01.012 PMC4089195

[27] Yates J , Perelman I , Khair S , et al. Exclusion criteria and adverse events in perioperative trials of tranexamic acid: a systematic review and meta‐analysis. Transfusion 2019;59(2):806‐824. 30516835 10.1111/trf.15030

[28] Sudhof LS , Shainker SA , Einerson BD . Tranexamic acid in the routine treatment of post‐partum hemorrhage in the United States: a cost‐effectiveness analysis. Am J Obstet Gynecol. 2019;221(3):275.e1‐275.e12. 31226298 10.1016/j.ajog.2019.06.030

[29] World Health Organization . WHO Recommendations on Prevention and Treatment of Postpartum Haemorrhage. Geneva, Switzerland: Word Health Organization; 2018 https://apps.who.int/iris/bitstream/handle/10665/75411/9789241548502_eng.pdf;jsessionid=D73E3FB9B3F78407FC8525D344D2AE1E?sequence=1. Accessed January 26, 2020.

[30] Franchini M , Mengoli C , Cruciani M , et al. Safety and efficacy of tranexamic acid for prevention of obstetric haemorrhage: an updated systematic review and meta‐analysis. Blood Transfus. 2018;16(4):329‐337. 29757132 10.2450/2018.0026-18 PMC6034773

[31] Novikova N , Hofmeyr GJ , Cluver C . Tranexamic acid for preventing postpartum haemorrhage. Cochrane Database Syst Rev. 2015;(6):CD007872. 26079202 10.1002/14651858.CD007872.pub3 PMC11236425

[32] Tranexamic acid for the prevention of obstetrical hemorrhage after cesarean (TXA) . ClinicalTrials.gov identifier: NCT03364491. https://clinicaltrials.gov/ct2/show/NCT03364491 Updated July 10, 2019. Accessed November 23, 2019.

[33] Sentilhes L , Lasocki S , Ducloy‐Bouthors AS , et al. Tranexamic acid for the prevention and treatment of postpartum haemorrhage. Br J Anaesth. 2015;114(4):576‐587. 25571934 10.1093/bja/aeu448

[34] Yang H , Zheng S , Shi C . Clinical study on the efficacy of tranexamic acid in reducing postpartum blood loss: a randomized, comparative, multicenter trial [in Chinese]. Chinese J Obstet Gynecol. 2001;36(10):590‐592. 16134519

[35] Gungorduk K , Asıcıoğlu O , Yildirim G , Ark C , Ismet Tekirdağ A , Besimoglu B . Can intravenous injection of tranexamic acid be used in routine practice with active management of the third stage of labor in vaginal delivery? A randomized controlled study. Am J Perinatol. 2012;30(5):407‐413. 23023559 10.1055/s-0032-1326986

[36] Mirghafourvand M , Mohammad‐Alizadeh S , Abbasalizadeh F , Shirdel M . The effect of prophylactic intravenous tranexamic acid on blood loss after vaginal delivery in women at low risk of postpartum haemorrhage: a double‐blind randomised controlled trial. Aust NZ J Obstet. 2015;55(1):53‐58. 10.1111/ajo.12262 25688820

[37] Sentilhes L , Winer N , Azria E , et al. Tranexamic acid for the prevention of blood loss after vaginal delivery. N Engl J Med. 2018;379(8):731‐742. 30134136 10.1056/NEJMoa1800942

[38] Sheldon W , Blum J , Vogel J , Souza J , Gülmezoglu A , Winikoff B . Postpartum haemorrhage management, risks, and maternal outcomes: findings from the World Health Organization Multicountry Survey on Maternal and Newborn Health. BJOG. 2014;121(suppl 1):5‐13. 24641530 10.1111/1471-0528.12636

[39] Nair M , Choudhury MK , Choudhury SS , et al. Association between maternal anaemia and pregnancy outcomes: a cohort study in Assam, India. BMJ Glob Health. 2016;1(1):e000026. 10.1136/bmjgh-2015-000026 PMC5321311 28588921

[40] Ker K , Roberts I , Chaudhri R , et al. Tranexamic acid for the prevention of postpartum bleeding in women with anaemia: study protocol for an international, randomised, double‐blind, placebo‐controlled trial. Trials. 2018;19(1):712. 30594227 10.1186/s13063-018-3081-x PMC6311062

[41] Al‐Zirqi I , Vangen S , Forsen L , Stray‐Pedersen B . Prevalence and risk factors of severe obstetric haemorrhage. BJOG. 2008;115(10):1265‐1272. 18715412 10.1111/j.1471-0528.2008.01859.x

[42] Gabel K , Lyndon A , Main E. Risk factor assessment. In: Lyndon A, Lagrew D, Shields L, Main E, Cape V Improving Health Care Response to Obstetric Hemorrhage, Version 2.0. A California Quality Improvement Toolkit. Stanford, CA: California Maternal Quality Care Collaborative; 2015:76‐79. https://www.cmqcc.org/resource/ob-hem-risk-factor-assessment. Accessed January 3, 2020.

